# Veratrum as a Specific in Treatment of Puerperal Eclampsia

**Published:** 1888-09

**Authors:** F. M. Rushing


### VERATRUM AS A SPECIFIC IN TREATMENT OF PUERPERAL ECLAMPSIA.

BY F. M. RUSHING, M.D.

CASE I.—Mrs. Hawkins, aged eighteen or twenty years, primipara, previous health tolerably good, some vertigo, and perhaps feet and legs slightly swollen, at full term of uterine gestation; was taken sick on the morning of the 11th day of April, 1881. At about 9 o'clock she had a fearful convulsion; Dr. J. G. Moore lived near by and was called in immediately. She continued having them every hour from then until after midnight. I arrived between 12 and 1 o'clock next morning. She was comatose and presented a most hideous appearance, her features were enormously swollen and tongue protruding out, and so badly swollen that it seemed impossible to get it back in her mouth. I made a digital examination and found the os but slightly dilated. I asked the doctor what he had done, and he said everything he could think of except venesection. I proposed the Norwood veratrum hypodermically. He said: "I am willing to try anything you may suggest." I charged my syringe with 7 drops Norwood's veratrum in a little water, and before I could get ready to use it, she had one of the most terrible convulsions I ever saw. As soon as she became sufficiently quiet I inserted the medicine and in a short time gave an enema of 100 grains of pot. brom. with 30 or 40 grains chloral in warm water. She had no more symptoms of convulsions and became conscious in the course of two hours. She was then given 30 or 40 grains brom. pot. every two hours, and when pulse would get up 3 or 4 drops veratrum with it. She was delivered naturally of a healthy male child between 10 and 11 o'clock, and made a rapid recovery.

CASE II.—Mrs. Harper, aged sixteen or seventeen years, primipara, anemic, feet and legs had been swollen, was at full term of pregnancy; commenced having pains about 1 o'clock, a.m., of the second day of June last; a midwife was sent for; at 5 o'clock in the morning she had a convulsion. Dr. J. W. Garrett was sent for immediately (residence about four miles off), he got there between 6 and 7 o'clock, and commenced giving chloroform by inhalation, and kept it up until I arrived there, between 1 and 2 o'clock, with no other effect than modifying or lessening the severity of fits. She had had six or eight convulsions, and was unconscious, her pains were weak, if she had any. I made a digital examination per vaginum and found the head pressing upon the perineum. We had a short consultation and agreed upon using the forceps; but upon a second digital examination I decided that I could, with proper manipulations, deliver without the forceps, and did so in a very short time.

The child was a healthy male. I at once proposed the veratrum hypodermically, stating to the doctor that she was subject then as before the delivery to have the fits, in which opinion he agreed. I decided as she had been delivered I would combine morphia and atropia with it.

| | | |
|---|---|---|
| ℞. | Veratrum, Norwood tinct | *m* vi, |
| | Morphia | gr. ⅙, |
| | Atropia | gr. 1-160. |
| | Aqua | q. s. *m* xxx. |

Before I could get ready she had a severe convulsion. I gave it before her jerkings went off. She then took veratrum 3 *m.*, pot brom. 20 grains, every two or three hours, she had no more convulsions, was peart next morning and got well without further treatment. I have treated several like cases with the same happy results, and mention these two dissimilar cases, to show that it does not make much difference whether they are plethoric or anemic, the results are the same, and strange to say, none of the cases have ever had any appearances of nausea. I shall not pretend to enter into the etiology of the disease or the manner in which the medicine acts specifically more than to state in my opinion, it calms the circulation, quiets nervous irritability, controls reflex action and destroys or modifies the poisonous action of the uric acid upon blood or brain. I do not claim any originality in this treatment, but do not now recollect of having ever seen any account of it before I commenced it.

